# Smiling, Yawning, Jaw Functional Limitations and Oral Behaviors With Respect to General Health Status in Patients With Temporomandibular Disorder—Myofascial Pain With Referral

**DOI:** 10.3389/fneur.2021.646293

**Published:** 2021-05-24

**Authors:** Joanna Kuć, Krzysztof Dariusz Szarejko, Maria Gołȩbiewska

**Affiliations:** ^1^Department of Prosthodontics, Medical University of Bialystok, Białystok, Poland; ^2^Private Health Care, Physical Therapy and Rehabilitation, Bialystok, Poland; ^3^Department of Dental Techniques, Medical University of Bialystok, Białystok, Poland

**Keywords:** anxiety, depression, health, jaw functional limitation, myofascial pain with referral, oral behaviors, orofacial pain, temporomandibular disorder

## Abstract

**Background:** The temporomandibular joint is the one of the most important joints in the human body. It enables numerous orofacial functions such as mastication, swallowing, breathing, speech, emotional communication, and facial expressions. The aim of the study was to evaluate the prevalence of jaw functional limitations and oral behaviors with respect to general health status in patients with temporomandibular joint disorders—myofascial pain with referral.

**Materials and methods:** The study group consisted of 50 individuals (37 females and 13 males) with complete natural dentition. The average age was 23.36 years with ± 0.30 as a standard error. All subjects underwent clinical examination and were diagnosed with myofascial pain with referral according to the Diagnostic Criteria for Temporomandibular Disorders. The survey was conducted in connection with the Jaw Functional Limitation Scale-8 (JFLS-8), Jaw Functional Limitation Scale-20 (JFLS-20), Patient Health Questionnaire-4 (PHQ-4), Patient Health Questionnaire-9 (PHQ-9), Generalized Anxiety Disorder-7 (GAD-7), Patient Health Questionnaire-15 (PHQ-15), and Oral Behaviors Checklist (OBC).

**Results:** The most common functional problems in the entire study group were chewing tough food and yawning. In terms of gender, statistically significant differences were noted for chewing tough food and smiling (*p* = 0.015451; *p* = 0.035978, respectively). With respect to Bonferroni correction and Benjamini-Hochberg procedure, the observed differences were not statistically significant. There were no statistically considerable differences in mastication, mandibular mobility, verbal and emotional communication, or global limitations (*p* > 0.05). Over half (56%) of the respondents had depression of varying severity. Somatic symptoms of different severity were found in 78% of the patients, and 44% of the respondents declared anxiety disorders. The score of the Oral Behavior Checklist (OBC = 27.18) highlighted a high tendency for developing craniomandibular disorders.

**Conclusion:** Patients with myofascial pain with referral, demonstrated a disturbed biopsychosocial profile. The restrictions in yawning and smiling as well as limitations in mastication, mobility, verbal and emotional communication, and global limitations appear to be significant predictors of craniomandibular dysfunction. Depression, stress, and somatic disorders are important factors predisposing patients to the occurrence of myofascial pain with referral. The progression of oral behaviors may indicate the role of somatosensory amplification.

## Introduction

Currently, due to the growing number of affected people, temporomandibular disorders are becoming a special disease in the field of dentistry as well as an important public health problem. The prevalence of such conditions is more common among females than males (1–3). The symptoms appear between 20 and 40 years of age, and the signs tend to worsen with age (1, 4).

Temporomandibular disorders are dysfunctions of multifactorial nature, embedded in the biopsychosocial model (5–7). The biomechanical theory suggests a relationship between functional load and capacity of the masticatory system (8). Its long-lasting overuse and/or overload may induce pain and dysfunction in people with decreased resistance within the musculoskeletal system (9, 10). Some authors emphasize that temporomandibular disorders are always related—directly or indirectly—to the myofascial component, i.e., trigger points (TrPs) of the head and neck (11). The contributing factors include micro- and retrogenia, cervical spine disorders, onychophagia, grinding or gnashing of the teeth, biting foreign objects, leaning on the hand, and continuous gum chewing (11–13). A significant role is attributed to the protraction of the head and shoulder girdle (11). Other potential risk factors include trauma, occlusal changes, and psychosocial factors (e.g., stress, coping strategies, anxiety, depression, catastrophizing, and emotional status) (14, 15).

According to the American Academy of Orofacial Pain (AAOP), myofascial disorders of the craniofacial region are characterized by dull, local pain which is aggravated by movements of the mandible (5, 16–18). Ailments occur in the face, jaws, temples, the pre-parotid region or in the ear area, at rest or during activities. Hyperactive spots or trigger points appear in a tense band of muscle tissue or fascia. Stimulation of these zones changes pain modulation, revealing the transferred pattern (5, 16–18). Myofascial pain may be accompanied by the feeling of muscle stiffness, subjective impression of bite decalibration without the possibility of clinical verification, tinnitus, dizziness, toothache, tension-type headaches, limited mouth opening and hyperalgesia in the area of transferred pain (5, 16–18). The extended protocol of the Diagnostic Criteria for Temporomandibular Disorders (DC/TMD) additionally includes the role of psychological, behavioral, and psychosocial factors (19). DC/TMD are based on the biaxial model. Axis I emphasizes the impact of pathobiological (physical) factors on the human body (6). Axis II points to the psychosocial, psychological and behavioral assessment of the patient, including jaw functional limitation, oral behaviors, anxiety, depression, and somatization (20).

The aim of the study was to evaluate the prevalence of jaw functional limitations and oral behaviors with respect to general health status in patients with temporomandibular joint disorders—myofascial pain with referral.

Considering that depression and anxiety are important components of biopsychosocial profile predisposing patients to the occurrence of myofascial pain with referral, it was hypothesized that at least one of the following variables—jaw functional limitations, somatization, anxiety and/or oral behavior–has a statistically significant relationship with depressive symptoms. It was also suggested that there will be statistically significant prediction of anxiety by jaw functional limitations, depression, somatization, and oral behavior.

## Materials and Methods

### Subjects and the Size of the Sample

The study group consisted of 50 people (37 females and 13 males) with complete natural dentition, who had been referred to the Department of Prosthodontics at the Medical University of Bialystok, Poland. The average age of the subjects was 23.36 years with ± 0.30 as a standard error. All of them underwent a clinical examination according to the Diagnostic Criteria for Temporomandibular Disorders (DC/TMD) (Axes I and II). The patients were diagnosed with myofascial pain with referral (Axis I of DC/TMD) (19).

#### Inclusion Criteria

- Craniofacial and/or craniomandibular pain of at least 8 points according to VAS (Visual Analog Scale);
- Full natural dental arches (Class I of Angle's Molar Classification, canine position);
- Lack of orthodontic history or retention status over 36 months after the treatment completion.

#### Exclusion Criteria

- Trauma within the craniofacial and/or craniomandibular area;
- Surgical treatment within the craniofacial and/or craniomandibular region;
- Dental therapy supported by an occlusal splint;
- Prosthetic treatment
- Physioterapeutic rehabilitation within the craniofacial and/or craniomandibular region;
- Cases with possible health concerns affecting the function of the masticatory muscles;
- Metabolic diseases;
- Drugs.

The above-mentioned subjects and sample size remain in accordance with the study group described in the previous publication (21, 22).

### Questionnaires With Respect to Axis II of DC/TMD

The following questionnaires associated with jaw functional limitations, oral behaviors, anxiety, depression, and somatization were evaluated:

- Jaw Functional Limitation Scale-8 (JFLS-8)
- Jaw Functional Limitation Scale-20 (JFLS-20)
- Patient Health Questionnaire-4 (PHQ-4)
- Patient Health Questionnaire-9 (PHQ-9)
- Generalized Anxiety Disorder-7 (GAD-7)
- Patient Health Questionnaire-15 (PHQ-15)
- Oral Behaviors Checklist (OBC) (19).

### Statistical Analysis

The Statsoft Statistica 13.3 software (TIBCO Software Inc., Statsoft, Cracow, Poland), G Power v.3.1.9.4 (Germany) and PQStat 1.8.2. (PQStat Software, Poznan, Poland) were used for statistical analyses. The arithmetic mean, median, and measures of differentiation involving standard deviation were calculated. The Mann–Whitney *U*-test was used to assess significant differences in the groups divided based on gender. A Pearson's chi-squared test of independence for a 2 × 2 contingency table was applied to compare categorical variables. In the case of small sample size, when the expected number of frequencies was below 5, a one-sided Fisher's exact test was used. All differences with *p* < 0.05 were considered statistically significant. For the one-sided Fisher's exact test, a *post-hoc* power analysis was conducted. Statistical power (1-β) was evaluated based on the calculation of the effect size, α and sample size (*n*). Additionally the sample size required to detect a statistically significant difference between females and males (at the 0.05 level) with a probability of 0.8 (80%) was determined.

A multiple-comparison correction was performed. To monitor the family-wise error rate and receive the Bonferroni critical value (Bonferroni adjusted *p*-value), *p* = 0.05 was divided by the number of tests (*n* = 10, Table 1; *n* = 21, **Table 6**). To monitor the false discovery rate the Benjamini–Hochberg procedure was conducted.

**Table 1:** Jaw functional limitations with respect to JFLS-8 in the entire study group (n = 50), the female group (n = 37) and the male group (n = 13).

											**Comparison with respect to gender**			
**JFLS-8**	**Reference value**	**Entire group n** **=** **50**			**Female group n** **=** **37**			**Male group n** **=** **13**			**Mann-Whitney U-test**		**Sample size for 80% test power**	**Benjamini-Hochberg correction**
		**Mean**	**±SD**	**Me**	**Mean**	**±SD**	**Me**	**Mean**	**±SD**	**Me**	**p-value**	**1-β**	**n**	**p-value**
Chewing tough food	0–10	3.78	3.01	4.00	4.38	2.88	4.00	2.08	2.81	1.00	0.02^*^	0.7789821	55	0.15
Chewing chicken (e.g., prepared in an oven)	0–10	1.76	2.67	0.00	2.00	2.74	1.00	1.08	2.43	0.00	0.13	0.2796107	270	0.27
Eating soft food without chewing (e.g., mashed potatoes, apple sauce, pudding, pureed food)	0–10	0.80	1.55	0.00	0.73	1.24	0.00	1.00	2.27	0.00	0.80	0.1142924	1,545	0.89
Opening the mouth wide enough to drink from a cup	0–10	1.22	2.08	0.00	1.24	1.99	0.00	1.15	2.41	0.00	0.41	0.0638498	20,295	0.59
Swallowing	0–10	0.70	1.71	0.00	0.59	1.42	0.00	1.00	2.38	0.00	0.98	0.1538447	775	0.98
Yawning	0–10	3.16	3.12	2.50	3.32	3.18	3.00	2.69	3.01	2.00	0.60	0.1498168	815	0.75
Talking	0–10	0.56	1.36	0.00	0.65	1.44	0.00	0.31	1.11	0.00	0.25	0.1962426	485	0.41
Smiling	0–10	0.76	1.65	0.00	0.57	1.63	0.00	1.31	1.65	0.00	0.04^*^	0.3830367	170	0.18
Sum of total points of all the above-mentioned aspects	0–80	12.74	12.48	9.50	13.49	11.32	12.00	10.62	15.68	4.00	0.12	0.1543106	770	0.27
Arithmetic average of total points (sum score of all items on the short form divided by number of items answered)	0–10	1.59	1.56	1.19	1.69	1.41	1.50	1.33	1.96	0.50	0.11	0.1550114	760	0.27

* *p < 0.05 statistical significance*.

A multiple linear regression models for PHQ-9 and GAD-7 estimation were developed by selecting the variables that contributed significantly to PHQ-9 and GAD-7 (GAD-7, PHQ-4, PHQ-15; PHQ-4, PHQ-9, respectively). No interactions with JFLS-8, JFLS-20, OBC were present in any of these analyses. In the case of GAD-7 no statistically significant relationship with PHQ-15 was noted.

### Ethical Approval

The study was conducted upon obtaining the consent of the Bioethics Committee of the Medical University of Bialystok (decision No. R-I-002/322/2016). The research was performed in accordance with the principles of the Declaration of Helsinki of the World Medical Association (WMA) as well as the Guidelines for Good Clinical Practice (GCP). Participation in the study was voluntary. All the patients received comprehensive information about the nature, scope of clinical activities and course of the proceedings. Every patient consented in writing to participate in the study. The subjects had the right to withdraw from the experiment at any time without any resulting consequences.

## Results

The most common functional problems in the entire study group were chewing tough food and yawning (JFLS-8) (Table 1). Slightly less frequently, patients reported limitations associated with eating roast chicken and opening their mouth wide. The average sum of JFLS-8 points in the entire study group was 12.74. The female group obtained 13.49 points, and the male group scored 10.62 (Table 1). With respect to gender, statistically significant differences were noted for chewing tough food and smiling (*p* = 0.015451; *p* = 0.035978, respectively) (Table 1). With regard to the Bonferroni correction and the Benjamini–Hochberg procedure, no statistically significant differences were observed (Table 1).

The mean value of the mastication index was 1.93 points in the entire study group (JFLS-20) (Table 2). In males, this parameter amounted to 1.69 points, while in the group of females it was 2.01. The average value of the mandibular mobility restriction was similar in the entire study group, in the group of females and in the group of males, and oscillated within 2 ± SD points. The verbal and emotional limitation amounted to 0.74 points in the entire study group. With respect to gender, this restriction was considerably higher in males than in females. In the entire study group, the level of global limitations was 1.61 points. In females this parameter amounted to 1.57 points, while in males the limitation was slightly higher and reached the value of 1.73 (Table 2). There were no statistically significant differences between the female and male group in terms of mastication, mandibular mobility, verbal and emotional communication or global limitations (*p* > 0.05; Table 2).

**Table 2:** Jaw functional limitations with respect to JFLS-20 in the entire group (n = 50), the female group (n = 37) and the male group (n = 13).

														**Comparison with respect to gender**		
	**Reference value**				**Entire group** ***n*** **=** **50**			**Female group** ***n*** **=** **37**			**Male group** ***n*** **=** **13**			**Mann–Whitney** ***U*****-test**		**Sample size for 80% test power**
**JFLS-20**	**Lack of TMD**		**TMD**													
	**Mean**	**±SD**	**Mean**	**±SD**	**Mean**	**±SD**	**Me**	**Mean**	**±SD**	**Me**	**Mean**	**±SD**	**Me**	**p-value**	**1-β**	**N**
Limitations in mastication	0.28	0.02	2.22	0.13	1.93	1.96	1.25	2.01	1.79	1.50	1.69	2.45	0.50	0.16	0.1151753	1,515
Limitations in mobility	0.18	0.02	2.22	0.13	2.16	2.34	1.13	2.08	2.03	1.50	2.38	3.13	0.75	0.93	0.0959059	2,605
Limitations in verbal and emotional communication	0.14	0.02	0.72	0.10	0.74	1.10	0.50	0.60	0.87	0.50	1.13	1.56	0.50	0.38	0.3475950	195
Global limitations	0.16	0.02	1.74	0.11	1.61	1.52	1.23	1.57	1.38	1.25	1.73	1.94	0.65	0.79	0.0867532	3,730

Limitations in mastication were found in 17 (34%) participants of the study, including 14 (38%) women and 3 (23%) men (Table 3). Restrictions in mandibular mobility were noted in 19 (38%) patients. Twenty (40%) subjects suffered from limitations in verbal and emotional communication. Global limitations were observed in 18 (36%) patients (Table 3). There were no statistically significant differences of TMD with regard to gender (*p* > 0.05; Table 3).

**Table 3:** The prevalence of limitations in mastication, mobility, verbal, and emotional communication and global restrictions with respect to JFLS-20 in the entire group (n = 50), the female group (n = 37) and the male group (n = 13).

					**Comparison with respect to gender**		
**JFLS-20**	Reference value^*^	**Entire group**	**Female group**	**Male group**	**Fisher's exact unilateral test**		**Sample size for 80% test power**
	**TMD**	**n = 50**	**n = 37**	**n = 13**	**p-value**	**1-β**	**n**
Limitations in mastication	>2.09–7.83	17 (34%)	14 (38%)	3 (23%)	0.27	0.1541494	345
Limitations in mobility	>2.09–10.00	19 (38%)	15 (41%)	4 (31%)	0.39	0.0889079	820
Limitations in verbal and emotional communication	>0.62–5.50	20 (40%)	15 (41%)	5 (38%)	0.58	0.0336680	18,025
Global limitations	>1.63–5.90	18 (36%)	14 (38%)	4 (31%)	0.46	0.0694843	1,295

* *Range of reference values calculated with respect to DC/TMD (23) and the presented study results*.

*%percentage within column (% entire group; % female group; % male group, respectively)*.

In relation to PHQ-4, 29 (58%) of the respondents did not declare any health problems (Table 4). Mild disorders were observed in 13 (26%) individuals, and moderate complaints were reported by 6 (12%) subjects. A severe health condition affected 2 (4%) patients, including 1 female and 1 male (Table 4). There were no statistically significant differences in the prevalence of PHQ-4 disorders based on gender (*p* > 0.05; Table 4).

**Table 4:** Health status with respect to the patient health questionnaires (PHQ-4, PHQ-9, PHQ-15) and generalized anxiety disorder (GAD-7) in the entire study group (n = 50), the female group (n = 37) and the male group (n = 13).

					**Comparison with respect to gender**					
**Questionnaires**	**Reference values**	**Entire group**	**Female group**	**Male group**	**Pearson chi-squared test (χ^**2**^** **test)**			**Fisher's exact unilateral test**		**Sample size for 80% test power**
		**n = 50**	**n = 37**	**n = 13**	**Chi^**2**^**	**df**	**p-value**	**p-value**	**1-β**	**n**
**PHQ-4**					**Normal vs. all others**					
Normal	0–2	29 (58%)	21 (57%)	8 (62%)	0.0902949	1	0.76	0.51	0.0489995	3,200
Mild	3–5	13 (26%)	11 (30%)	2 (15%)						
Moderate	6–8	6 (12%)	4 (11%)	2 (15%)						
Severe	9–12	2 (4%)	1 (3%)	1 (8%)						
**PHQ-9**					**None vs. all others**					
None	0–4	22 (44%)	15 (41%)	7 (54%)	0.6912007	1	0.41	0.31	0.1214343	510
Mild	5–9	18 (36%)	14 (38%)	4 (31%)						
Moderate	10–14	7 (14%)	6 (16%)	1 (8%)						
Moderately severe	15–19	2 (4%)	2 (5%)	0 (0%)						
Severe	20–27	1 (2%)	0 (0%)	1 (8%)						
**PHQ-15**					**Minimal vs. all others**					
Minimal	0–4	11 (22%)	10 (27%)	1 (8%)	–	–	–	0.14	0.2775750	150
Low	5–9	21 (42%)	12 (32%)	9 (69%)						
Medium	10–14	14 (28%)	12 (32%)	2 (15%)						
High	15–30	4 (8%)	3 (8%)	1 (8%)						
**GAD-7**					**None to minimal vs. all others**					
None to minimal	0–4	28 (56%)	20 (54%)	8 (62%)	0.2187002	1	0.64	0.45	0.0695543	1,295
Mild	5–9	15 (30%)	13 (35%)	2 (15%)						
Moderate	10–14	6 (12%)	4 (11%)	2 (15%)						
Severe	15–21	1 (2%)	0 (0%)	1 (8%)						

*% percentage within column (% entire group; % female group; % male group, respectively)*.

More than half (56%) of the respondents had symptoms of depression of varying severity (PHQ-9) (Table 4). Its mild stage was reported in 36% of cases. Moderate depression was observed in 14% of the participants, including 6 females and 1 male. A moderately severe condition was found in 4% of the cases (Table 4). No statistically significant differences were observed with respect to gender (*p* > 0.05; Table 4).

Low-severity somatic symptoms (PHQ-15) were noted in 21 (42%) subjects (Table 4). Medium-level disorders were recorded in 14 (28%) patients, including 12 (32%) females and 2 (15%) males. Somatic symptoms of high intensity were found in 4 (8%) patients including 3 females and 1 male (Table 4). There were no statistically significant differences with respect to gender (*p* > 0.05; Table 4).

Anxiety disorders of varying severity (GAD-7) were declared by 22 (44%) respondents (Table 4), among whom mild-stage concerns were observed the most often (30% of patients). Moderate dysfunction were found in 6 (12%) subjects, including 4 (11%) females and 2 (15%) males. Severe anxiety was reported by 1 (8%) male patient. No statistically significant differences were demonstrated with respect to gender (*p* > 0.05; Table 4).

Multiple linear regression model revealed that GAD-7, PHQ-4 and PHQ-15 questionanires allowed the differentiation of about 88% PHQ-9 cases (*R*^2^ = 0.88422775), and the prediction model was significantly better than the random one [*F*_(3_, _46)_ = 117.11; *p* < 0.00], as in the former the average error in evaluating the level of PHQ-9 was SE = 1.5693 (Table 5).

**Table 5:** Multiple linear regression model with the PHQ-9 as the dependent variable and GAD-7, PHQ-4, and PHQ-15 as independent variables.

	**Regression coefficient (b)**	**SE**	**Standardized coefficient (β)**	**t-value**	**p-value**	**Tolerance score**	**R^**2**^-value**	**Semipartial correlations (r)**	**Durbin-Watson statistic**
Intercept	0.04725	0.48630	-	0.09716	0.92	–	–	–	
GAD-7	0.41062	0.11453	0.39492	3.58510	0.00^*^	0.207413	0.792587	0.179856	1.731663
PHQ-4	0.60215	0.15865	0.39262	3.79540	0.00^*^	0.235190	0.764810	0.190406	
PHQ-15	0.26273	0.06541	0.25699	4.01702	0.00^*^	0.614934	0.385066	0.20154	

*R = 0.94033385*.

*R^2^ = 0.88422775*.

*Adjusted R^2^ = 0.87667739; [F_3, 46_ = 117.11 p < 0.00]*.

*Standard error of the estimate: 1.5693*.

*SE, standard error*.

* *p < 0.05 statistical significance*.

**Figure 1:**
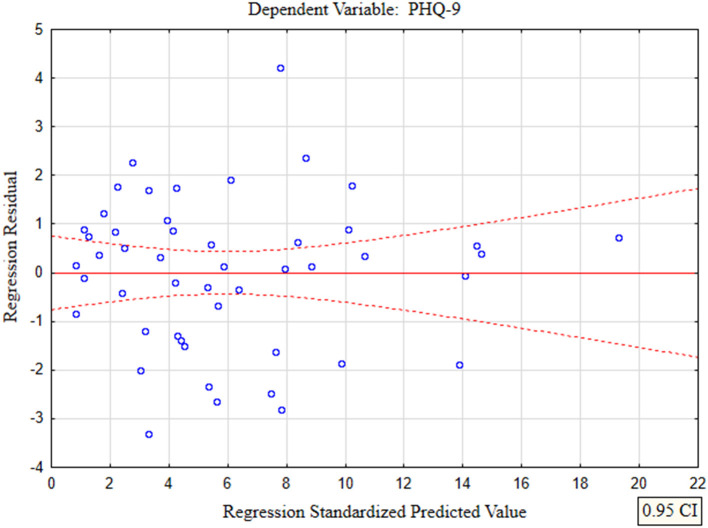
The plot of standardized residues vs. standardized predicted values (homoscedasticity) with respect to multiple linear regression model for PHQ-9 estimation.

**Figure 2:**
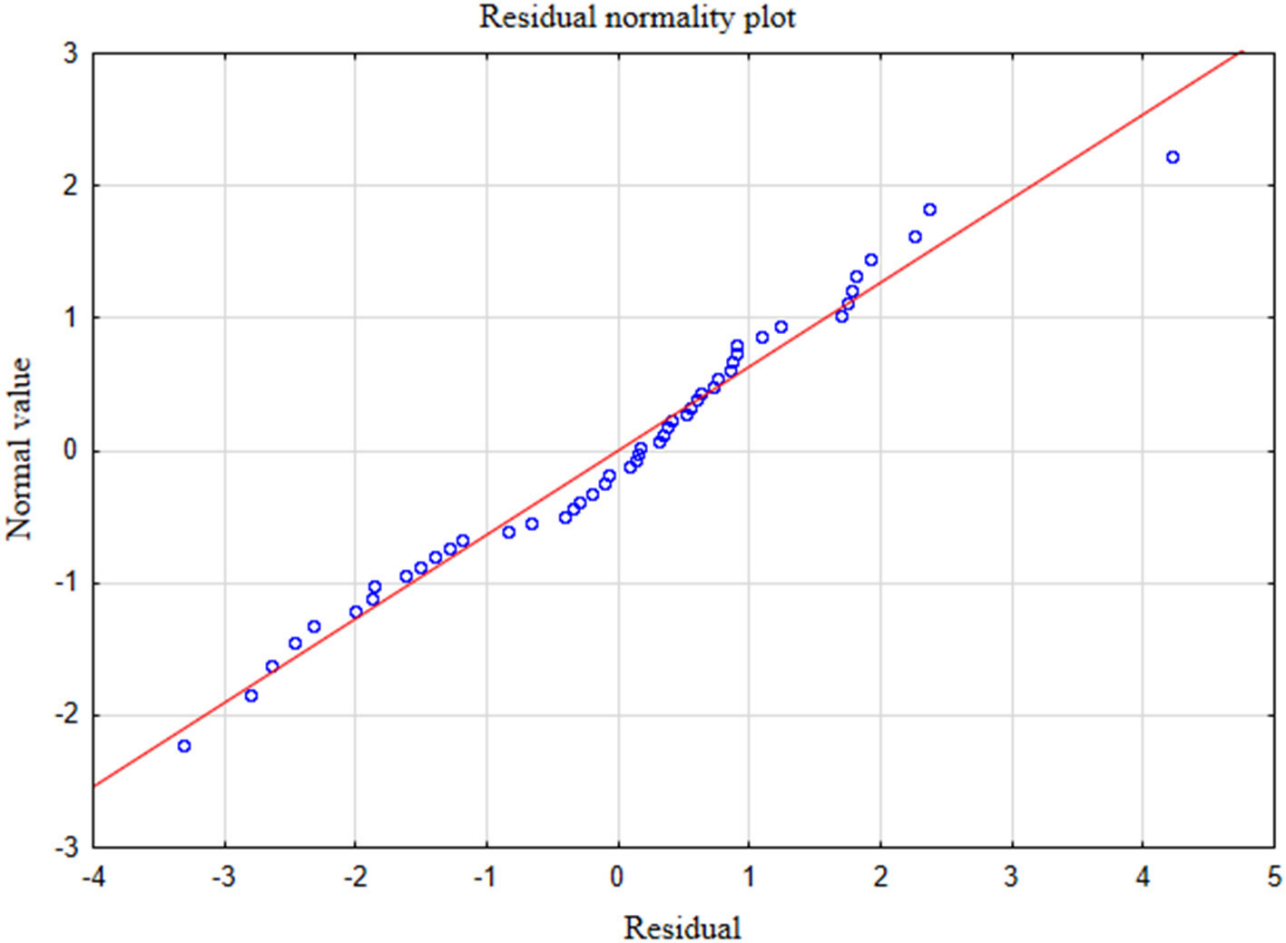
Normality of the distribution of residuals with respect to multiple linear regression model for PHQ-9 estimation.

In the presented regression model, the first assumption regarding linearity was met and the equation of multiple regression was statistically significant [*F*_(3, 46)_ = 117.11; *p* < 0.00; *R* = 0.94033385] (Table 5). The criteria of the statistical significance of partial regression coefficients of GAD-7, PHQ-4 and PHQ-15 were also fulfilled (*p* = 0.00; Table 5). The third assumption about the lack of multicollinearity could be violated. This was confirmed by the obtained tolerance scores (GAD-7 = 0.207413, PHQ-4 = 0.235190, PHQ-15 = 0.614934) and *R*^2^-values (GAD-7 = 0.792587, PHQ-4 = 0.764810, PHQ-15 = 0.385066). Semipartial correlations confirmed weak link between GAD-7, PHQ-4 and PHQ-15 with PHQ-9 (*r* = 0.179856, *r* = 0.190406, *r* = 0.20154 respectively). The next fourth assumption about homoscedasticity was also met (Figure 1). The fifth requirement for the lack of residual autocorrelation was fulfilled (Durbin–Watson = 1.731663) (Table 5). Sixth assumption about the normality of the distribution of residuals could be violated (Figure 2). Due to the lack of extreme deviations, this study results are still valid. All Cook's distance values were below 1.0. It means that individual cases did not have an excessive effect on the model.

The second model of multiple linear regression revealed that PHQ-4 and PHQ-9 questionanires enabled the differentiation of about 84% GAD-7 cases (*R*^2^ = 0.83779373), and the prediction model was significantly better than the random one [*F*_(2_, _47)_ = 121.38; *p* < 0.00], as in the former the average error in evaluating the level of GAD-7 was SE = 1.7674 (Table 5).

**Figure 3:**
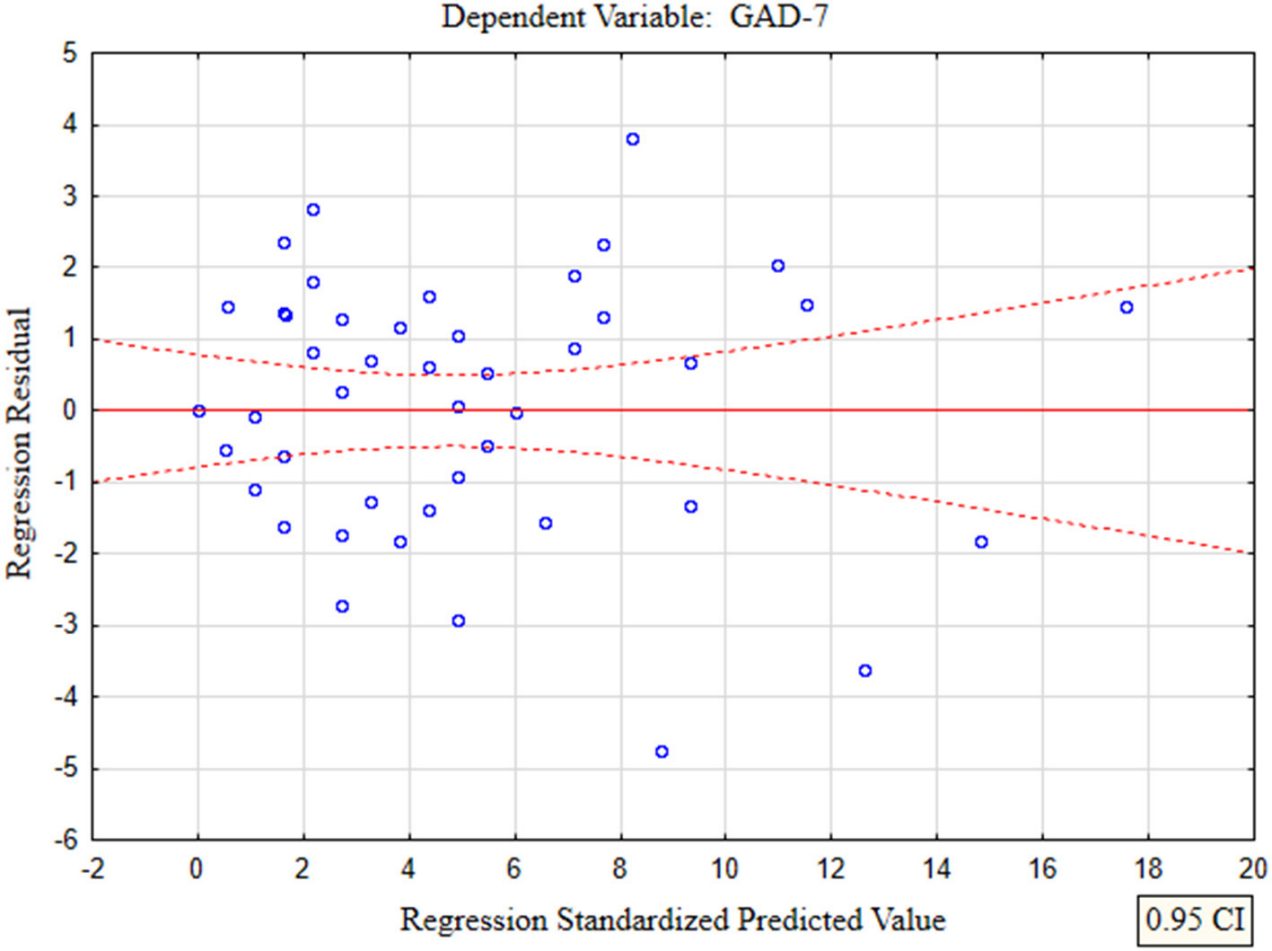
The plot of standardized residues vs. standardized predicted values (homoscedasticity) with respect to multiple linear regression model for GAD-7 estimation.

**Figure 4:**
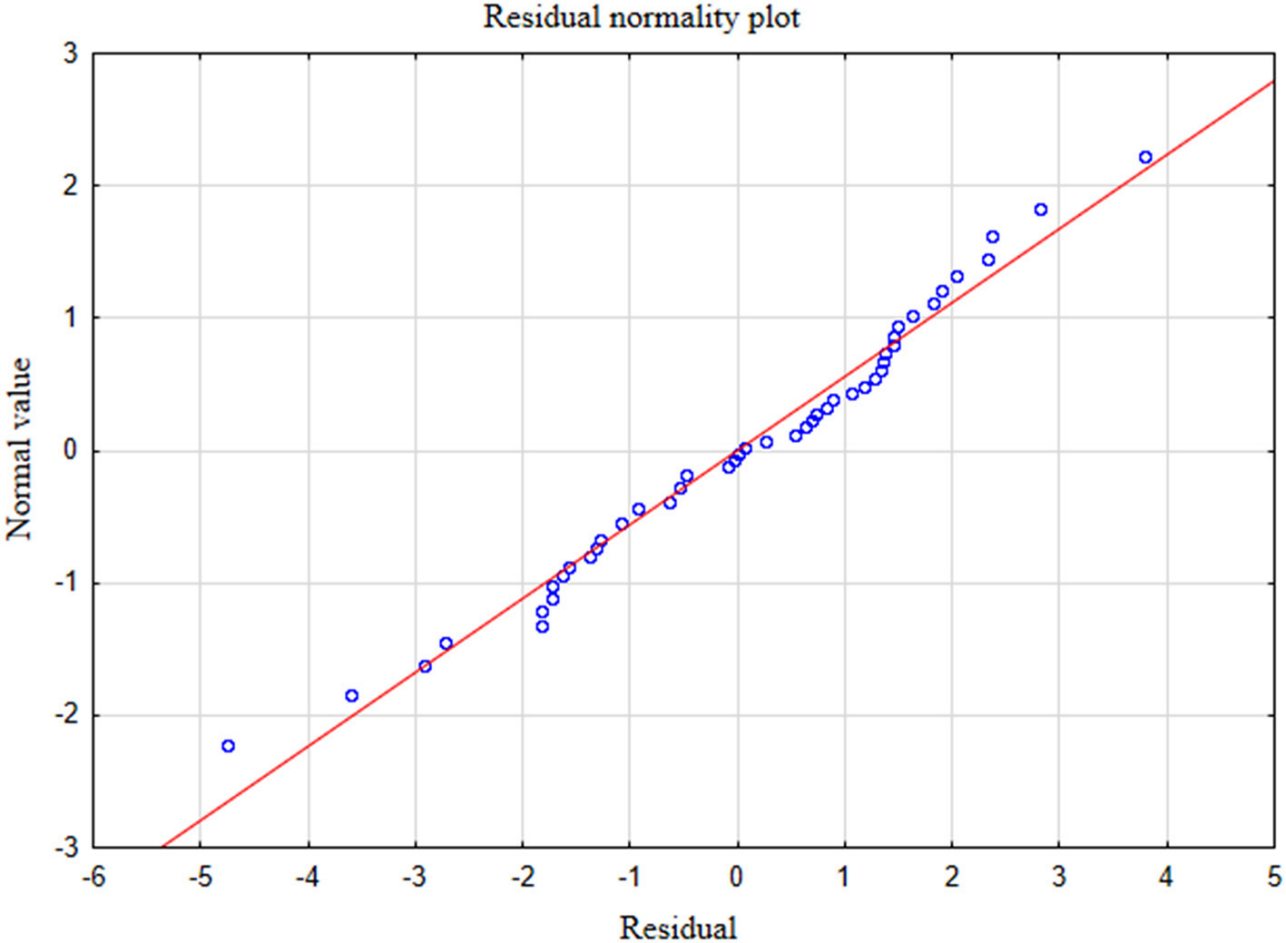
Normality of the distribution of residuals with respect to multiple linear regression model for GAD-7 estimation.

In the presented regression model, the first assumption about linear relationship between predictor variables (PHQ-4, PHQ-9) and outcome variable (GAD-7) was fulfilled. The multiple regression equation was statistically significant [*F*_(2, 47)_ = 121.38; *p* < 0.00; *R* = 0.91531073] (Table 6). The second assumption about statistical significance of partial regression coefficients of PHQ-4 and PHQ-9 was also met (p = 0.00; Table 6). The third assumption about the lack of multicollinearity (redundancy) between independent variables could be violated. This was confirmed by the obtained tolerance scores (PHQ-4 = 0.226836, PHQ-9 = 0.226836) and *R*^2^-values (PHQ-4 = 0.773164, PHQ-9 = 0.773164) (Table 6). Semipartial correlations confirmed low relationship between PHQ-4 and PHQ-9 with GAD-7 (*r* = 0.178798, *r* = 0.270324, respectively) (Table 6). The plot of standardized residues vs. standardized predicted values showed no obvious signs of a funnel suggesting that the variance of the residuals is constant (the fourth assumption about homoscedasticity) (Figure 3). The next, fifth assumption about the lack of residual autocorrelation was also met. The Durbin-Watson statistic was close to 2.0 (Durbin–Watson = 1.683588) (Table 6). The sixth assumption about the normality of the distribution of residuals could be violated (Figure 4). Due to the fact that only extreme deviations from normality could have a significant impact on the findings, this study results are still valid. The seventh assumption about the lack of influential cases biasing the regression model was fulfilled. All Cook's distance values were below 1.0, which suggests that individual cases did not have an excessive effect on the model.

**Table 6:** Multiple linear regression model with the GAD-7 as the dependent variable and PHQ-4 and PHQ-9 as independent variables.

	**Regression coefficient (b)**	**SE**	**Standardized coefficient (β)**	**t-value**	**p-value**	**Tolerance score**	**R^**2**^-value**	**Semipartial correlations (r)**	**Durbin-Watson statistic**
Intercept	−0.01121	0.42993	–	−0.02607	0.98	–	–	–	
PHQ-4	0.55375	0.18194	0.37541	3.04352	0.00^*^	0.226836	0.773164	0.178798	1.683588
PHQ-9	0.54588	0.11863	0.56758	4.60150	0.00^*^	0.226836	0.773164	0.270324	

*R = 0.91531073*.

*R^2^ = 0.83779373*.

*Adjusted R^2^ = 0.83089134; [F_2, 47_ = 121.38 p <0.00]*.

*Standard error of the estimate: 1.7674*.

*SE – standard error*.

* *p < 0.05 statistical significance*.

The Oral Behavior Checklist (OBC) revealed that the most common problem in the entire study group was sleeping in positions causing pressure on the mandible (Table 7). The average value of this symptom amounted to 3.38 points. In terms of gender, the observed data were comparable. The intensity of clenching or grinding of the teeth during sleep was determined at 2.14 points, while the results for yawning, clenching of the teeth during daily activity, pressing, touching and keeping teeth in contact in a manner different than while eating, unilateral mastication and eating between meals that required chewing ranged from 1.6 to 1.84 points. The average sum of points from the entire questionnaire obtained in the study group was 27.18. In females, this parameter amounted to 27.59 points, and in males it oscillated near the value of 26 points (Table 7). There were no statistically significant differences with respect to gender (*p* > 0.05; Table 7).

**Table 7:** Oral behaviors with respect to the oral behavior checklist in the entire study group (n = 50), the group of females (n = 37) and the group of males (n =13)–Axis II of DC/TMD.

											**Comparison with respect to gender**			
		**Entire group n** **=** **50**			**Female group n** **=** **37**			**Male group n** **=** **13**			**Mann–Whitney U-test**		**Sample size for 80% test power**	**Benjamini-Hochberg correction**
**OBC-20**	**Reference values**	**Mean**	**±SD**	**Me**	**Mean**	**±SD**	**Me**	**Mean**	**±SD**	**Me**	**p-value**	**1-β**	**n**	**p-value**
✓Clenching or grinding of the teeth while sleeping	0–4	2.14	1.60	2.00	2.19	1.60	2.00	2.00	1.68	2.00	0.75	0.0969753	2,510	0.94
✓Sleeping in a position that induces pressure on the mandible (e.g., on the stomach, on the side)	0–4	3.38	1.21	4.00	3.41	1.24	4.00	3.31	1.18	4.00	0.45	0.0810379	4,935	0.81
✓Grinding the teeth together during waking hours	0–4	0.74	1.07	0.00	0.68	1.03	0.00	0.92	1.19	1.00	0.42	0.1584495	730	0.81
✓Clenching the teeth together during waking hours	0–4	1.68	1.20	2.00	1.81	1.15	2.00	1.31	1.32	1.00	0.18	0.3304161	210	0.81
✓Pressing, touching, or holding the teeth together in situations other than eating	0–4	1.80	1.18	2.00	1.70	1.20	2.00	2.08	1.12	2.00	0.34	0.2523179	320	0.81
✓Holding, tightening or tensing muscles without clenching or bringing the teeth together	0–4	1.34	1.22	1.00	1.32	1.16	2.00	1.38	1.45	1.00	0.97	0.0657275	16,120	0.99
✓Holding or jutting the jaw forward or to the side	0–4	0.72	1.09	0.00	0.62	0.98	0.00	1.00	1.35	0.00	0.39	0.2473404	330	0.81
✓Pressing the tongue forcibly against the teeth	0–4	0.78	1.07	0.00	0.68	1.00	0.00	1.08	1.26	1.00	0.22	0.2760077	275	0.81
✓Placing the tongue between the teeth	0–4	1.00	1.36	0.00	1.00	1.37	0.00	1.00	1.35	0.00	0.90	–	–	0.99
✓Biting, chewing or playing with the tongue, cheeks, or lips	0–4	1.04	1.23	0.50	0.97	1.17	0.00	1.23	1.42	1.00	0.61	0.1473030	845	0.91
✓Holding the jaw in a rigid or tense position, such as to brace or protect the mandible	0–4	1.42	1.23	1.00	1.49	1.22	1.00	1.23	1.30	1.00	0.47	0.1517506	795	0.81
✓Biting or holding objects (such as hair, a pipe, a pencil, a pen, fingers, fingernails etc.) between the teeth	0–4	0.80	1.09	0.00	0.86	1.13	0.00	0.62	0.96	0.00	0.50	0.1682060	645	0.81
✓Using chewing gum	0–4	1.44	1.23	1.50	1.46	1.26	2.00	1.38	1.19	1.00	0.92	0.0735424	7,900	0.99
✓Playing musical instruments that involves the use of the mouth or jaw (e.g., woodwind, brass, string instruments)	0–4	0.00	0.00	0.00	0.00	0.00	0.00	0.00	0.00	0.00	–	–	–	–
✓Leaning with the hand on the jaw, such as cupping or resting the chin in the hand	0–4	1.46	1.18	1.00	1.46	1.10	1.00	1.46	1.45	1.00	0.76	–	–	0.94
✓Chewing food on one side only	0–4	1.64	1.31	1.50	1.81	1.31	2.00	1.15	1.21	1.00	0.11	0.4673261	130	0.81
✓Eating between meals (eating food that requires chewing)	0–4	1.84	1.13	2.00	1.95	1.13	2.00	1.54	1.13	1.00	0.28	0.2872645	260	0.81
✓Sustained talking (e.g., teaching, sales, customer service)	0–4	1.10	1.34	1.00	1.16	1.46	1.00	0.92	0.95	1.00	0.99	0.1438985	890	0.99
✓Singing	0–4	0.52	0.81	0.00	0.54	0.90	0.00	0.46	0.52	0.00	0.71	0.0934503	2,845	0.94
✓Yawning	0–4	1.60	1.01	1.00	1.68	1.08	1.00	1.38	0.77	1.00	0.48	0.2451952	330	0.81
✓Holding a telephone between the head and shoulders	0–4	0.74	0.92	0.00	0.81	0.97	0.00	0.54	0.78	0.00	0.42	0.2330646	360	0.81
Sum of total points of all above-mentioned aspects	0–84	27.18	10.65	23.00	27.59	10.05	23.00	26.00	12.58	20.00	0.35	0.1097587	1,730	0.81

***Reference value for the risk of temporomandibular disorders (TMD):** Sum of OBC (23): 0, no risk; 1–24, low risk; 25–84, high risk; Sum of OBC (23): 0–16, normal behaviors; 17–24, score occurs twice as often in those with TMD, 25–62, score occurs 17 times more often; only a result in the range of 25–62 causes TMD*.

## Discussion

Temporomandibular disorders are connected with multiple clinical manifestations. They may lead to functional limitations of the masticatory system and/or psychosocial conditions including reduced quality of life (24). The most common difficulties involve limited chewing efficiency and/or unilateral mastication (25–27). Typically observed phenomena also include disturbances of rhythm, strength and pattern of the chewing cycle as well as discoordination and limitation of mandibular mobility (28). During mastication, inappropriate recruitment of the temporal and masseter muscles appears on both working and balancing sides (28–30). The severity of functional orofacial problems might depend on the choice of food, consumption habits and pleasure of eating. A lot of patients with temporomandibular joint disorder modify their diet. A strong link has been observed between avoidant eating behavior and progression of TMD as patients tend to eliminate certain kinds of food due to their texture and/or consistency. This may in turn affect the nutritional status (30–34).

In the presented study, the main restriction was chewing tough food (JFLS-8) (Table 1). Normative value for chewing tough food (JFLS-8) in the general population in Sweden was 1.23 (35). The mean value for this limitation in the entire study group amounted to 3.78 points. This could result from the overload of the masticatory system, which indicates that food of greater density requires strong biting forces as well as greater vertical and lateral mandibular movements with repetitive TMJ motion (30). Haketa et al. reported that patients with myofascial pain demonstrate fewer difficulties chewing than those with disc displacement, either with or without reduction (25).

Interestingly, the second major restriction in the entire study group was related to yawning (Table 1). The yawning index was 6.32 times higher than that observed by Oghli et al. in the Swedish population (35). Some authors highlight that yawning exacerbates pain. This is probably the effect of involuntary rapid jaw movements which affect disc position and result in quick stretch of the masticatory muscles and structures of TMJ. The consequences might be biting imbalance and loss of joint control (36). Furthermore, morning yawning may release intra-articular adhesions with all their typical consequences.

The lack of possibility of spontaneous yawning might lead to various clinical outcomes. Yawning includes the pandiculation of the masseter, temporal and pterygoid muscles and the prolonged contraction of the submandibular muscles (37). It is usually connected with the pandiculation of other muscles in the body which benefit from muscle elongation (37). This phenomenon is defined as the stretch-yawning syndrome (SYS) (37, 38). There are two kinds of pandiculation. The first one is associated with the extension of the trunk and limbs when the flexors are elongated and extensors are contracted. In the second type the opposite happens—the extensors are elongated and the flexors are contracted (37).

In the study on an animal model, Bertolucci suggested that pandiculation and yawning play a significant role in the autoregulation of the locomotor system (38). It means that coordinated and integrated body movements could be conditioned by regular resetting and restoring functional and structural balance within the myofascial system (38). It is probable that the SYS facilitates an appropriate myofascial tonus which is necessary for muscle activity against gravity (38).

Breathing while yawning leads to pressure variations within the ventricular system inside the brain (39). After each deep inhalation, cerebrospinal fluid flow rate increases in the fourth ventricle (39). Wide mouth opening and inhalation influence intracranial circulation as an immediate consequence of cervical compression of jugular vessels. Vertical movements of the mandible activate the pterygoid musculovenous pump. The mechanism of cranial venous blood flow is accelerated via the venous plexus of the foramen ovale (39). A musculovenous motor chain reaction occurs in a form of tonic waves to the skeletal muscles as well as ends of the limbs—fingers and toes (39). The SYS results in increased activity of the parasympathetic nervous system (38), whereas yawning stimulates structures responsible for cortical activation (39). Thus, SYS inhibition may cause disturbances in the human body homeostasis. Over time, the incidence of disorders, particularly musculoskeletal, could be expected (38). In the presented study group, this potential consequence could be reflected in the prevalence of orofacial and general pain location demonstrated in the previous publication (21) and reported yawning factor at the level of 3.16 points (Table 1).

Restrictions of yawning and pandiculation could lead to disturbances in the clearance of somnogenic substances such as prostoglandin (PGD 2), thus intensifying sleepiness (39). In turn, decreased activity of the parasympathetic system could result in a large number of parasympatethic dysfunctions with all functional consequences for the entire body (40). It should be mentioned that parasympatethic stimulation of appropriate receptors leads to: constriction of the pupil (miosis), improvement of near vision, erection in males, decreased heart rate and velocity of conduction through the atrioventricular node, vasodilation, upward tendency of bronchial secretions, increased secretion of potassium ions, water and amylase within the salivary glands, increased motility and relaxation of sphincters within the stomach and intestines, increased rate of gastric secretions and gallbladder stimulation, release of digestive enzymes and insulin, stimulation of ureteral peristalsis, contraction of the detrusor muscle, and relaxation of the internal urethral sphincter (40). Moreover, yawning-related reduction in the activity of the parasympatethic nervous system is associated with the burnout syndrome that includes emotional exhaustion, negative perception of work and the feeling of lack of competence in the performance of tasks required by the employer (41). Typically, also affective, physical, cognitive, and behavioral symptoms are observed (41). Provine emphasized that yawning, as well as sneezing and coughing lead to often underestimated secondary consequences, including changes in mood, attention and state of arousal (42).

Another significant problem in the presented study group was difficulty smiling (JFLS-8) (Table 1). Rychlowska et al. emphasized that smiles of reward, affiliation and dominance are responsible for basic social functions such as rewarding behavior, creating social bonds and negotiating hierarchy (43). These authors pointed out that multifaceted nature of human smile could help express multiple social intentions and emotions, especially love, sympathy, and war (43). Mancini et al. highlighted that a major factor in non-verbal communication and a social skill crucial for the development and maintenance of interpersonal relationships is the ability to interpret emotions and differentiate them from facial expressions (44). This poses a question of whether problems with smiling may trigger any kind of social withdrawal. Smile is a powerful tool that positively affects the human body in numerous aspects. People who smile genuinely and naturally are perceived as kinder and more sociable, honest, pleasant, careless, and polite (45). The intensity of smiling also affects the perception of warmth and competence of humans (45). In the study on the Swedish population, Oghli et al. showed that restrictions related to smiling were at the level of 0.5 points (35). In our research this limitation amounted to 0.76 points.

Smiling is connected with mirroring facial expressions and emotions. The mirror neuron system consists of neurons activated both when people perform certain specific motor actions and when they only recognize similar acts in others (46). It means that positive and negative emotions can be evoked unconsciously. Furthermore, significant aspects of emotional face-to-face communication occur similarly on an unconscious level (47). Navarretta emphasized that facial expressions are mirrored also in situations dealt with for the first time (46). Emotional copying behavior is a frequent and extremely important phenomenon in both interpersonal face-to-face communication and social life. Navarretta demonstrated that smiling and laughing are the most frequently mirrored behaviors (60 and 48% of the occurrence, respectively) (46), which means that one person's smile makes other people smile back (46). Marmolejo-Ramos et al. concluded their study with the statement: “your face and moves seem happier when I smile” (48). Our facial expressions and moves tend to be more cheerful in response to other people's smile. However, the potential role of anti-mirror neurons should not be forgotten in such relationships (49). Difficulty smiling should draw our attention to alexithymia (50). Von Piekartz et al. demonstrated that facial emotion recognition is disrupted in people with chronic pain (50). The authors stated that a possible cause of this phenomenon could be deficits in cortical motor processing rather than cortical emotion processing (50). Bearing in mind the entire spectrum of musculoskeletal disorders—particularly myofascial pain with referral—it could be suggested that in the presented study group smile mirroring was impossible, as is observed in the course of alexytymia.

In relation to JFLS-20, restrictions typical of TMD, i.e., concerning mastication, mandibular motion, emotional and verbal expression, as well as global limitations occurred in about or slightly over 40% of the respondents, respectively (Tables 2, 3). Ohrbach et al. demonstrated that in studies conducted on four groups of patients—with temporomandibular joint disorders (I), primary Sjögren's syndrome (II), malocclusion (III) and oral burning syndrome (IV)—the greatest restrictions of chewing and mandibular mobility appeared in Group I (24). In the case of emotional and verbal limitations, the results in Group I and Group III were comparable, with slightly higher values obtained in Group I (24). Recent studies revealed a significant inversely proportional relationship between JFLS-20 and cervical range of motion in patients with TMD as well as in those with both TMD and cervicogenic dizziness (51). The authors suggested the existence of a direct link between TMD and cervical spine impairment (51). In the general population in Sweden, Oghli et al. showed a statistically significant relationship between oral and general health status and jaw functional limitation scores (35). The authors found that the health status worsened when JFLS scores increased (35).

Ohrbach et al. emphasized a strong correlation between JFLS-20 and JFLS-8 (r > 0.94) (24). The authors declared that in patients with temporomandibular joint disorders, the evaluation of links between limitations in mastication, emotional/verbal expression and both JFLS scales yielded in comparable values of correlation coefficients of above 0.8 points (24). On the other hand, in terms of restrictions in mandibular mobility, a stronger correlation coefficient was noted for JFLS-20 (*r* > 0.83) than for JFLS-8 (*r* > 0.68) (24). Because in patients with temporomandibular joint disorder, the most common complaints are related to the mobility of the mandible, it is more appropriate to use JFLS-20 (24). Ohrbach et al. stressed that, due to the global factor, the extended scale of functional restrictions (JFLS-20) is completely satisfactory, while JFLS-8 has certain limitations (24). However, in the latest research, 96% of the tested physical therapists reported that JFLS-8 used together with physical tests is a proper tool in TMD asessment (52), although it should be mentioned that the interpretation of the results obtained from JFLS-8 has not been established yet (23).

It has been observed that the prevalence of depression in patients with chronic pain amounts to 30–54% (53), and that females suffer more often than males (53). The incidence rate for gender is 1.5:2.1 (53). Some authors point out that depression raises the threshold of feeling chronic pain (53, 54). Other studies indicate the lack of statistically significant differences in the severity of chronic pain between patients diagnosed with depression and those who do not suffer from it (55). It is also well-known that depression often conditions the expression of temporomandibular joint disorders (56).

In relation to the DC/TMD criteria, the verification of mental anxiety or depressive symptom is based on PHQ-4 and PHQ-9 questionnaires (23, 57–59). In the presented study, 42% of the respondents suffered from mental health problems (PHQ-4) (Table 4). Depressive symptoms (PHQ-9) were found in 56% of the patients (Table 4). The results of this study indicate a significant role of mental instability in patients with masticatory dysfunction. Fear and anxiety trigger the sympathetic part of the autonomic nervous system, leading to accelerated pulse, increased muscle tone, excessive sweating from the apocrine glands, as well as behavioral disorders, i.e., quick speech or body tremor (60). Anxiety intensifies central and peripheral effects associated with pain (60). Ohrbach et al. found a statistically significant link between JFLS-8 and depression confirmed by the Symptom Checklist-90 (SCL-90) (*p* = 0.02) (61). Moreover, these authors observed no relationship between anxiety and somatization or any characteristics and interference of pain (61). Xu et al. in turn, showed a weak correlation between depression, anxiety, and JFLS-20 (62). These researchers observed a slight relationship in the case of somatization as well as a moderate link regarding pain (62).

Somatization, i.e., a tendency to perceive non-specific physical symptoms, corresponds to PHQ-15 (23, 63, 64). This condition is characterized by mental discomfort expressed as pain of unknown origin, palpitations, shortness of breath, tremor of the limbs, nausea, vomiting, abdominal pain, a tendency to faint, and a number of other ailments (65). In the presented study, somatic symptoms of a varying severity were found in 78% of the patients (Table 4). Regarding GAD-7, anxiety disorders of various intensity affected 44% of the subjects (Table 4) (66). The relationship between the PHQ-9 and the PHQ-4, PHQ-15, and GAD-7 was expressed by the multiple linear regression model (Table 5).

Reiter et al. stressed that depression symptoms are more pronounced in patients with chronic temporomandibular joint pain than in those with its acute phase (67). Furthermore, in subjects with masticatory dysfunction, depression and somatization are definitely intensified in cases with greater disability expressed as a high level of pain according to GCPS (the Graded Chronic Pain Scale) (67). Regarding chronic temporomandibular joint diseases, the GAD-7 questionnaire may be of less importance than those associated with depression and somatization (53, 67). However, the results of our study revealed the interplay between GAD-7 and both PQ-4 and PQ-9 (Table 6).

All of the above-mentioned data indicate that most patients with a disturbed biopsychosocial component of Axis II of DC/TMD require interdisciplinary treatment. It appears advisable to implement cognitive and behavioral methods. Cognitive methods consist of the techniques of distraction, re-evaluation of thoughts, mindfulness, pros and cons, continuity, and daily record of success. Behavioral methods include breath control, relaxation, activity, behavioral changes and experiments, actions aimed at solving problems directly and pharmacology (60).

Other important factors leading to temporomandibular disorders are parafunctions (68, 69). The presence of improper oral behaviors suggests central deregulation manifested by excessive motor activity, insufficient central nervous system inhibition, proprioception impairment and/or persistent excessive psychophysiological reactivity (70). In the entire study group, the global coefficient of perioral behaviors was 27.18 points. In females, this parameter amounted to 27.59 points, while in the group of males it was 26.00 (Table 7). These scores highlighted a high tendency for developing craniomandibular disorders (23). The most common reasons for this condition in the entire study group were clenching or grinding of the teeth during sleep as well as sleeping in positions that cause pressure on the mandible, e.g., on the stomach or on the side (Table 7). In healthy Portuguese individuals, Barbosa et al. showed that behaviors associated with sleeping positions were at the level of 3.3 points (71). In our research the incidence of this parafunction amounted to 3.38 points. In the case of clenching and grinding of the teeth while sleeping, results noted by Barbosa et al. were 0.74 points lower than our results (Table 7) (71). These authors demonstrated that the general sum score of OBC in their study reached 20 points (71). In our research, this parameter was 7.18 points higher (Table 7). In presented study other parafunctions concerned clenching of the teeth during daily activities, touching of the teeth and keeping them in contact under circumstances other than eating (Table 7). Additional risk factors included unilateral chewing and eating between meals (Table 7).

Some authors suggest that oral behaviors remain in a strong relationship with somatosensory amplification that involves hypervigilance of the body. It refers to the selective focus on experienced sensations and increased concentration on oneself (72, 73). Conversely, bodily hypervigilance could be connected with occlusal hypervigilance, including repetitive bite checking (74). From this point of view specific oral behaviors involving tooth-to-tooth interactions, tongue-to-teeth contact and/or clenching of the teeth might enable monitoring possible intraoral threats (73). Chow et al. noted a link between bodily and occlusal hypervigilance and the prevalence of oral behaviors. These researchers highlighted that somatosensory amplification tends to be greater in patients with facial pain than in pain-free cases (73). However, recent studies revealed no relationship between severity of somatosensory amplification and the frequency of oral behaviors and trait anxiety (75).

The temporomandibular joint is the one of the most important joints in the human body. It enables numerous orofacial functions such as mastication, swallowing, breathing, speech, emotional communication and facial expressions. Due to multiple potential causes of orofacial dysfunction, it is advisable to involve multidisciplinary treatment in the comprehensive evaluation and therapy of patients with temporomandibular disorders–myofascial pain with referral.

## Conclusion

Patients with myofascial pain with referral demonstrate a disturbed biopsychosocial profile manifested in a form of disorders of Axis II of DC/TMD. Restrictions in chewing tough food, yawning and smiling seem to be significant predictors of craniomandibular dysfunction. Myofascial pain with referral is connected with limitations in mastication, mobility, verbal and emotional communication as well as global limitations according to JFLS-20. Nearly 40% of patients with myofascial pain with referral suffer from jaw functional restrictions. Depression, stress and somatic disorders analyzed in relation to PHQ-4, PHQ-9, PHQ-15 and GAD-7 are important factors predisposing patients to myofascial pain with referral. The progression of oral behaviors may indicate the role of somatosensory amplification. Patients with myofascial pain with referral and an impaired biopsychosocial profile require multifaceted interdisciplinary treatment.

## Data Availability Statement

All relevant data is contained within the article.

## Ethics Statement

The studies involving human participants were reviewed and approved by the Bioethics Committee of the Medical University of Bialystok, Poland (decision No. R-I-002/322/2016). The patients/participants provided their written informed consent to participate in this study.

## Author Contributions

JK developed and planned the study. JK and KS conducted the research, contributed to sample preparation, and took the lead in writing the manuscript. MG contributed to the interpretation of the results and supervised the project. All authors discussed the results and contributed to the revision of the manuscript, read, and approved the submitted version.

## Conflict of Interest

The authors declare that the research was conducted in the absence of any commercial or financial relationships that could be construed as a potential conflict of interest.
